# Feasibility and acceptability of a remotely delivered, home-based “exercise snacking” to improve physical function in community-dwelling older adults: a 28-day pilot study

**DOI:** 10.3389/fmed.2026.1755508

**Published:** 2026-01-23

**Authors:** Zhongzhong Hu, Shuhuan Li, Xiaolong Shi, Keke Huang, Hao Huang, Xiaoyi Yuan

**Affiliations:** 1 School of Sports Science, Wenzhou Medical University, Wenzhou, China; 2 School of Education, Beijing Sport University, Beijing, China; 3 The First Affiliated Hospital of Wenzhou Medical University, Wenzhou, China; 4 Capital University of Physical Education and Sports, Beijing, China

**Keywords:** acceptability, exercise snacking, feasibility, home-based exercise, lower-limb strength, older adults, physical function

## Abstract

**Background:**

Age-related declines in muscle strength and physical function increase the risk of frailty, falls, and loss of independence in older adults. Traditional structured exercise programs often have low participation because of time constraints, limited access to facilities, and health-related barriers. “Exercise snacking” defined as brief bouts of resistance exercise accumulated across the day, may offer a more flexible and feasible option.

**Methods:**

In this 28-day pilot randomized controlled trial, 36 community-dwelling older adults aged 65–80 years were randomly assigned to a home-based “exercise snacking” (ES) group (*n* = 18) or a control group (*n* = 18). The ES group performed two daily bouts of a five-exercise, chair- and body weight based resistance circuit (~9 min per bout), remotely supervised via smartphone video submission, while controls continued their usual activities. Feasibility was assessed by retention and adherence (completed vs. prescribed sessions), and acceptability by a five-point Likert scale and semi-structured interviews. Physical function outcomes included the Short Physical Performance Battery [SPPB; 4-m walk, five-times sit-to-stand (5-STS), and a balance test], as well as the 60-s sit-to-stand (60 s-STS) and timed up and go (TUG) tests. Two-way repeated-measures analysis of variance and partial η^2^ was used to explore preliminary intervention effects.

**Results:**

Thirty-three participants (ES = 16, control = 17) completed follow-up (retention 88.9%). Among ES completers, mean adherence was 89.1%, with 49.9 ± 3.7 of 56 prescribed sessions completed. Acceptability was high (mean enjoyment 4.3/5), and 88.9% of participants reported intending to continue similar exercises independently. One minor adverse event (plantar fasciitis) and four mild musculoskeletal incidents were recorded, none leading to withdrawal. Compared with controls, the ES group showed significant improvements in SPPB total score, five-STS time, 4-m walk time, TUG time, and 60 s-STS repetitions, all with large effect sizes, while balance scores remained stable in both groups.

**Conclusion:**

A remotely delivered, home-based “exercise snacking” program appears feasible, acceptable, and safe for community-dwelling older adults and elicits meaningful short-term improvements in lower-limb strength and endurance, and gait speed. These findings support exercise snacking as a practical, low-burden strategy to counteract functional decline in aging populations and warrant larger, longer-term trials.

## 1 Introduction

The accelerating pace of global population aging has made the prevention of age-related declines in muscle function a major public health priority (1, 2). Sarcopenia, a syndrome characterized by progressive loss of muscle mass, strength, and physical performance, has emerged as a key risk factor that compromises older adults' quality of life, independence, and life expectancy (3, 4). This functional deterioration manifests not only as diminished ability to perform daily tasks, such as walking or rising from a chair, but also as increased risks of falls, disability, and hospitalization, placing a substantial burden on individuals, families, and healthcare systems (5, 6).

Regular physical exercise is widely recognized as one of the most effective behavioral interventions for mitigating age-related muscle decline (7). Among various exercise modalities, resistance training serves as the cornerstone for improving muscle mass, strength, and overall physical function (8). However, traditional structured exercise programs often require fixed schedules, designated facilities, or specialized equipment. Combined with mobility limitations, fragmented daily routines, and psychological barriers commonly found in older adults, these factors collectively contribute to persistently low participation rates (9, 10). Therefore, developing a more flexible, feasible, and time-efficient exercise model has become an urgent need in promoting healthy aging.

The emerging concept of “exercise snacking” addresses this need by dividing daily physical activity into multiple brief bouts of light to moderate intensity exercise, each lasting only a few minutes and easily incorporated into daily routines (11). Preliminary studies involving younger populations under laboratory conditions have demonstrated its beneficial effects on metabolic health (12–15). Perkin et al. (16) found that after 4 weeks of remote exercise snack supervision, healthy older adults exhibited significant improvements in 60 s sit-to-stand repetitions, reduced five-times sit-to-stand time, and increased thigh muscle cross-sectional area. Similarly, Western et al. observed that 4 weeks of exercise snacks led to reductions in timed up and go time and increases in the duration of left-leg single-leg balance. Liang et al. (17), extending the intervention to 12 weeks, found that exercise snacks positively affected lower-limb muscular endurance and single-leg balance in frail elderly individuals. In contrast, several studies (18–20) reported that exercise snacks did not significantly improve the 30 s/60 s sit-to-stand repetitions or five-times sit-to-stand time in frail elderly populations after 4 weeks.

The present study was designed as a pilot randomized controlled trial. Rather than seeking definitive therapeutic conclusions, it aimed to assess the feasibility (including adherence, safety, and acceptability) of a 4-week home-based exercise snacking intervention among community-dwelling older adults aged 65–80 years. In addition, the study sought to provide preliminary estimates of effect size for potential improvements in lower-limb strength, balance, and mobility, thereby informing future large-scale confirmatory trials. As a pilot investigation, the observation of positive trends in muscle function, even in the absence of statistical significance, would provide encouraging preliminary evidence for the value of exercise snacking in older adults. Ultimately, this study aims to explore a promising and highly feasible exercise strategy to counteract functional decline and support independent living in the aging population.

## 2 Materials and methods

### 2.1 Study design

This study employed a 4-week, pre-post, pilot randomized controlled trial to evaluate the feasibility and potential effects of a home-based resistance “exercise snacking” intervention on physical function in community-dwelling older adults. The trial was conducted in Beijing, China, between October and November 2025. All participants completed a survey on living conditions and underwent physical function assessments at a community senior activity center. The intervention was performed at home under remote supervision by the research team. Ethical approval for the study was obtained from sports science experiments of Beijing Sport University (2025489H).

### 2.2 Participant recruitment and screening

A total of 36 community-dwelling older adults aged 65–80 years who were not engaged in regular, structured exercise were recruited through posters distributed by community residents' committees and senior activity centers, as well as through referrals from community staff. Eligible participants were nonsmokers with a body mass index (BMI) of 20–30 kg/m^2^, free from contraindications to exercise or recent musculoskeletal injuries, and had access to a smartphone with a stable internet or Wi-Fi connection. During initial screening, participants were required to have a short physical performance battery (SPPB) score between 7 and 10, with no score of zero on any subcomponent. Exclusion criteria were as follows: (1) participation in structured resistance training more than once per week within the previous 3 months; (2) presence of acute or terminal illness likely to affect participation; (3) unstable or ongoing cardiovascular, metabolic, or respiratory disorders; (4) current use of insulin or corticosteroids affecting skeletal muscle metabolism; (5) musculoskeletal or neurological disorders limiting voluntary movement; and (6) inability to comply with study procedures or requirements. All eligible individuals provided written informed consent and were randomly allocated to the intervention or control group following completion of baseline assessments.

### 2.3 Exercise intervention

Participants in the exercise snacking group were instructed to perform two bouts of “exercise snacking” per day for 4 weeks during which time they did not engage in any additional exercise. Each bout comprised five exercises, performed for 1 min each, with participants encouraged to complete as many repetitions as possible within the allotted time. A 1-min rest period was provided between exercises, resulting in a total session duration of ~9 min. Participants were instructed to perform the exercises at home and to do so only when another person was present who could call for help in case of an emergency. The five exercises included: (1) sit-to-stand (STS) from a chair, (2) seated knee extensions with alternating legs, (3) standing knee bends with alternating legs, (4) marching in place, and (5) standing calf raises. Detailed demonstrations and safety precautions for these exercises are provided in Supplementary file A. For safety, participants were advised to hold onto a chair for support during standing exercises if needed. Participants were required to record themselves performing each session, positioning their smartphone at waist height to ensure the entire exercise was clearly and steadily captured. Videos were to be submitted to the research team before 10:00 p.m. on the day of completion. If a video was not received by the end of the day, researchers contacted the participant by phone to confirm whether the exercise session had been completed. Control group participants were instructed to maintain their usual lifestyle and were provided with all intervention materials after completing the final 4-week assessment.

### 2.4 Feasibility of the exercise intervention

Feasibility of the exercise intervention was evaluated based on participant retention and adherence to the intervention protocol. Retention was defined as the proportion of participants who were randomized and completed both the 4-week intervention and all follow-up assessments. Adherence was calculated as the number of completed sessions divided by the total number of prescribed sessions. In addition, adherence was further examined by comparing prescribed vs. completed: (1) number of exercise days per week, (2) frequency of “exercise snacks” per week, and (3) total number of “exercise snacks” completed during the 4-week intervention. The intervention was considered feasible if participant retention was at least 85% and the average adherence rate reached 80% or higher. The feasibility of the remote home exercise program was evaluated through pre-intervention home visits, which were conducted to identify potential barriers, including the availability of chairs with appropriate height for exercises and the adequacy of available space within the home.

### 2.5 Acceptability of the exercise intervention

Following the 4-week intervention, participants were invited to participate in a post-intervention interview to share feedback on their experiences and to evaluate the acceptability of the prescribed exercises. After their final training session, participants received written information describing the purpose of the interview and were reminded that participation was entirely voluntary. Interviews were conducted either by telephone or in person, according to participant preference. All interviews were digitally recorded, transcribed verbatim, and anonymized upon completion. Participants rated their enjoyment of the resistance “exercise snacking” program on a five-point Likert scale (1 = “not at all,” 2 = “a little,” 3 = “moderately,” 4 = “a lot,” 5 = “a great deal”) and indicated whether they intended to continue performing resistance “exercise snacking” independently at home. In addition, participants responded to open-ended questions regarding aspects they liked or disliked about the program. Those who did not intend to continue similar exercises after the intervention were asked to explain their reasons and suggest potential modifications to increase the likelihood of sustained engagement.

### 2.6 Adverse events and incidents

In this study, an adverse event referred to any exercise-related occurrence that led to a participant's temporary withdrawal from, or required adjustment of, the intervention program. An adverse incident was defined as a minor exercise-related issue, such as muscle or joint soreness or stiffness that did not necessitate interruption or modification of the prescribed exercises. All reported adverse events and incidents were promptly followed up by members of the research team. Participants were contacted by telephone to collect detailed information and to determine appropriate next steps, including whether they should continue the program with necessary adjustments or discontinue the exercises and seek medical evaluation.

### 2.7 Physical function assessments

Body height and weight were obtained using a TANITA WB-380H device (Tokyo, Japan) while participants were barefoot. A trained research psychologist evaluated each participant's capacity to safely engage in exercise snacking based on subjective judgments of balance stability and self-reported confidence in completing the assessment. The primary indicator of physical performance followed the short physical performance battery (SPPB) protocol (21), consisting of a balance assessment, gait speed evaluation, and a five-time sit-to-stand (five-STS) test. The SPPB yields a composite score between 0 and 12, where higher values indicate superior lower-limb performance. Two trials were completed, and the best performance was used for analysis. Scores are categorized as follows: 10–12, high function; 7–9, moderate limitation; 4–6, severe limitation; and 0–3, very severe limitation.

The balance assessments evaluated participants' ability to maintain stability across three stance conditions (side-by-side, semi-tandem, and tandem positions). Participants were instructed that they could use their arms, bend their knees, or adjust their body position to preserve balance but should avoid moving their feet. If a participant is unable to maintain a position for the full 10 s, testing does not proceed to the next stance. Scoring ranges from 0 to 4 points: 0 points are assigned if the side-by-side stance cannot be held for 10 s; 1 point if the side-by-side stance is held for at least 10 s but the semi-tandem stance is not; 2 points if the semi-tandem stance is held for at least 10 s but the tandem stance is held for < 3 s; 3 points if the tandem stance is maintained for 3 to 9.99 s; and four points if the tandem stance is successfully held for the full 10 s. For the five-STS test, participants started in a seated position on a standard chair with their arms crossed over the chest. They were instructed to rise to a full standing position and return to sitting five times as rapidly as possible. Performance was quantified as the total time required to complete all five sit-to-stand movements, measured from the moment the participant first left the chair until they were fully seated after the fifth repetition. For the gait speed assessment, participants were instructed to walk at their normal, comfortable pace along a straight 5-m walkway. The time required to traverse the central 4 m was measured with a stopwatch. To ensure an accurate and steady pace, participants began walking from a standing start positioned 0.5 m before the start line and continued 0.5 m beyond the finish line.

Secondary functional outcomes included the 60-s sit-to-stand (60 s-STS) test and the timed up and go (TUG) test. In the 60 s-STS, participants were instructed to perform as many sit-to-stand repetitions as possible within 60 s from a hard-backed chair with a seat height of 40 cm. Participants were allowed to use their arms for balance but not to assist movement from the chair. To minimize self-pacing, participants were positioned so that a clock was not visible, and repetitions were not counted aloud by the researcher. The TUG test was conducted to evaluate functional mobility. Participants began seated in a standard armchair with their back against the backrest. Upon the verbal cue “Go,” they stood up, walked at a comfortable and safe pace to a marker 3 m away, turned around, walked back, and sat down. Time in seconds was measured from the verbal cue until the participant's back touched the chair upon returning. Two trials were completed, and the best performance was used for analysis. The purpose of all assessment indicators is shown in Table 1.

**Table 1:** Purpose of the assessment.

**Assessments indicators**	**Purpose of the assessments**
4-m walk time	To assess walking speed and gait efficiency
Five-times sit-to-stand time	To assess lower-limb muscle strength and power during repeated sit-to-stand transitions
SPPB balance assessments	To assess static balance control and postural stability in progressively challenging standing positions
60-s sit-to-stand	To assess lower-limb muscular endurance and the ability to sustain repeated functional movements
Timed up and go time	To assess overall functional mobility, including integrating balance, walking ability and turning performance

### 2.8 Statistical analysis

As this investigation served as a preliminary trial, 36 older individuals were selected using a convenience sampling approach, and no prior computation of sample size was performed. Descriptive statistics were employed to summarize feasibility indicators such as participant retention and intervention compliance. Outcome measures were evaluated via a two-way repeated-measures analysis of variance (ANOVA). In instances where significant interactions between group and time appeared, Holm-Bonferroni adjustments were utilized for *post hoc* analysis. Statistical significance was determined at the *p* < 0.05 threshold. The magnitude of the intervention effect was quantified using partial eta squared (η^2^). The magnitude of the intervention effect was quantified factors, and provides a reliable estimate of the intervention effect in small sample sizes. Effect sizes are reported as point estimates. In accordance with Cohenmple sizese of the intervention effect was quantifiect, η^2^ ≈ 0.06 a medium effect, and η^2^ ≈ 0.14 a large effect. Results are expressed as means with standard deviations. All ANOVA and *post hoc* computations were executed using SPSS (version 26.0; IBM Corp., Chicago, IL, USA).

## 3 Results

**Figure 1:**
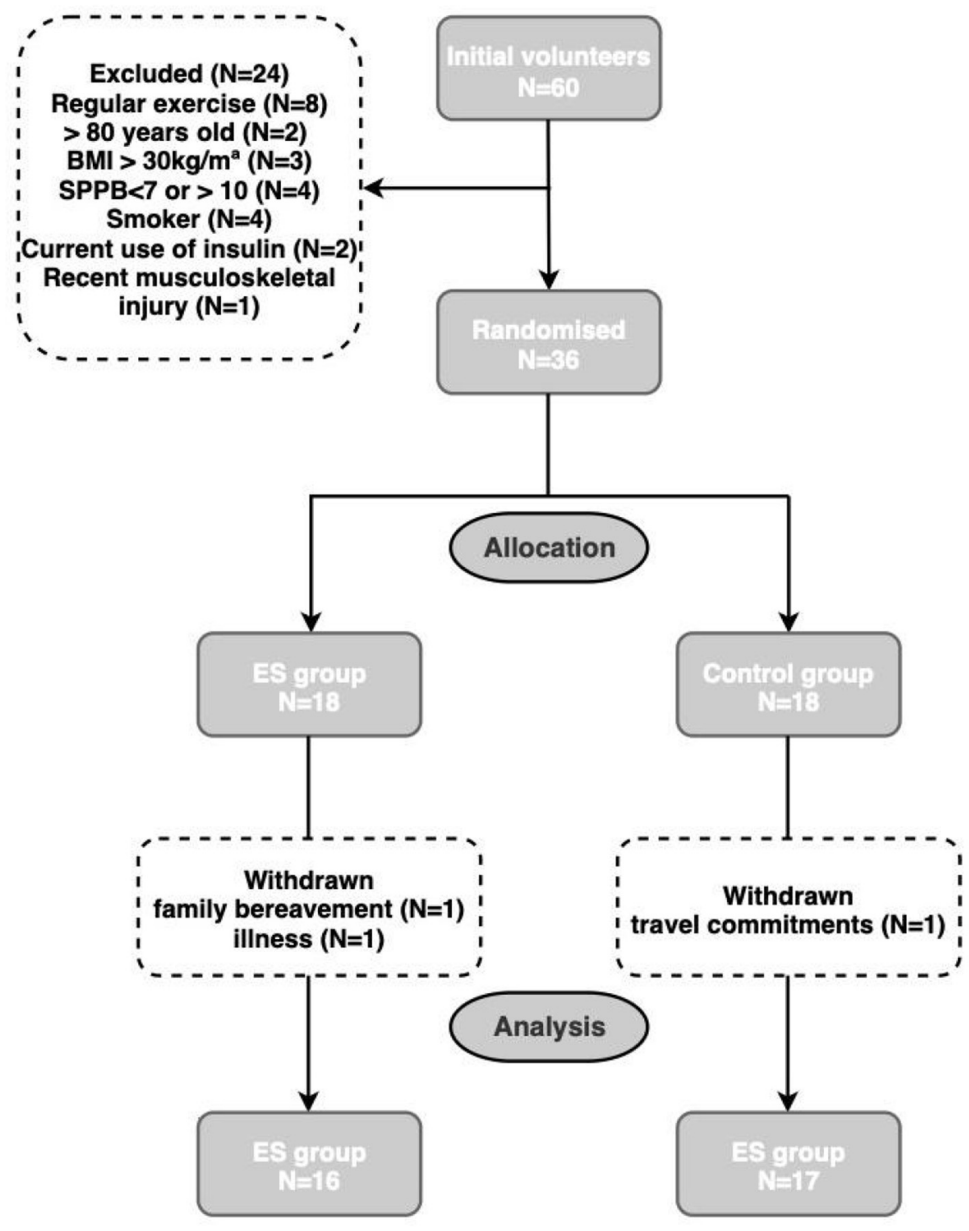
Flow diagram of participation throughout the study.

Figure 1 illustrates participant progression throughout the trial. Among the 60 individuals who responded to the recruitment advertisements, 36 successfully met the screening criteria and completed the follow-up evaluations. Participants were randomly assigned to either the intervention group (*n* = 18) or the control group (*n* = 18). Baseline demographic and physical function data for participants included in this pilot trial are presented in Table 2. No statistically significant differences were detected between the two groups in demographic or functional measures, indicating balanced allocation. The sample included both male and female participants and encompassed an age range of 65–80 years. A total of 33 participants completed the intervention phase and contributed outcome data at follow-up. Three participants withdrew from the study: two from the ES group and one from the control group. One participant in the ES group withdrew following a family bereavement and another discontinued due to illness. The participant in the control group was unavailable for follow-up because of travel commitments.

**Table 2:** Baseline characteristics of participants.

**Participant characteristics**	**Control (*N* = 18)**	**ES (*N* = 18)**
Male/female, *n*	8/9	10/6
Age (years), mean ± SD	72.7 ± 4.6	73.2 ± 5.0
Height (cm), mean ± SD	166.5 ± 8.7	167.2 ± 9.2
Weight (kg), mean ± SD	70.8 ± 12.6	71.7 ± 13.8
BMI (kg/m^2^), mean ± SD	25.5 ± 3.2	25.6 ± 3.4
**Marital status**, ***n*** **(%)**		
Unmarried	0	0
Married	12	11
Divorced	1	2
Widowed	4	3
Separated	1	2
**Employment**, ***n*** **(%)**		
Retired	11	11
Re-employ after retirement	1	2
Employed part-time	2	2
Self-employed	4	3
**Previous medical history**, ***n*** **(%)**		
Osteoporosis, *n*	3	2
Osteoarthritis, *n*	2	1
Hip replacement, *n*	1	0
Knee replacement, *n*	0	1
Rotator cuff surgery, *n*	1	0
Lumbar disc herniation, *n*	1	2
Adhesive capsulitis, *n*	0	1
History of fracture	1	1
**Physical function**		
SPPB total score	8.3 ± 0.8	8.4 ± 0.8
60-s STS (reps)	17.8 ± 4.9	17.4 ± 4.4
TUG time (s)	12.7 ± 2.2	12.5 ± 1.9

### 3.1 Feasibility and acceptability of the intervention

Based on the training video logs submitted by 16 participants who completed the follow-up assessments, individuals in the intervention group engaged in an average of 25.5 training days out of the planned 28, performing at least one session daily. The mean (SD) number of “exercise snacking” sessions undertaken was 49.9 (3.7) out of a potential total of 56. Among those who completed the intervention, overall retention reached 88.9% (16 of 18 participants), and overall adherence reached 89.1% (49.9 of 56 sessions), with three participants attending every scheduled session. Further details regarding adherence comparing prescribed and completed sessions are presented in Table 3, covering (i) weekly exercise frequency, (ii) the number of “exercise snack” sessions per week, and (iii) the total count of “exercise snack” sessions during the 4-week program.

**Table 3:** Adherence to the ES intervention presented as the prescribed vs. actual (completed) for: (i) number of days exercised per week, (ii) number of ES per week, and (iii) total number of ES during the 4-week intervention.

**Days exercised per week**		**Number of exercise sessions per week**		**Total number of exercise sessions during 4-week**	
**Prescribed**	**Actual mean (SD)**	**Prescribed**	**Actual mean (SD)**	**Prescribed**	**Actual mean (SD)**
7	6.4 (0.4)	14	12.5 (0.9)	56	49.9 (3.7)

Quantitative findings showed that participants rated the exercise snacking intervention highly, with a mean acceptability score of 4.3 out of 5, indicating strong overall approval. Consistently, qualitative interview data reflected generally positive attitudes toward the intervention. Approximately 88.9% of participants reported that they intended to continue performing similar resistance-based exercise snacking independently after the study. Regarding intervention acceptability, several prominent themes emerged, summarized along with illustrative quotations in Supplementary file A. Participants emphasized that the brief format of 9 min twice a day was practical and easily incorporated into their regular routines, requiring no sacrifice of other daily activities. Nevertheless, missed sessions were primarily attributed to factors such as vacations, social commitments, household responsibilities, illness, childcare duties, or demanding schedules. Many participants noted that establishing a consistent exercise time each day could support adherence and reduce the likelihood of skipping sessions. Feedback also indicated that tailoring exercise selection to individual skill levels and preferences may enhance future interventions. For instance, some participants expressed interest in greater variety, additional upper-body movements, and the use of external resistance.

### 3.2 Adverse events and incidents

One participant (1/18; 5.5%) in the exercise group reported a single adverse event during the intervention, identified as plantar fasciitis. The event was minor in severity and did not necessitate withdrawal from the program; the participant continued training with minor modifications, such as reducing repetitions in specific exercises. In addition, three participants (3/16; 18.75%) in the exercise group reported a total of four minor adverse incidents throughout the intervention. These incidents primarily involved mild musculoskeletal symptoms, such as stiffness or soreness in the thigh, calf, or ankle, which did not hinder continued participation in the training sessions.

### 3.3 Outcome data

**Figure 2:**
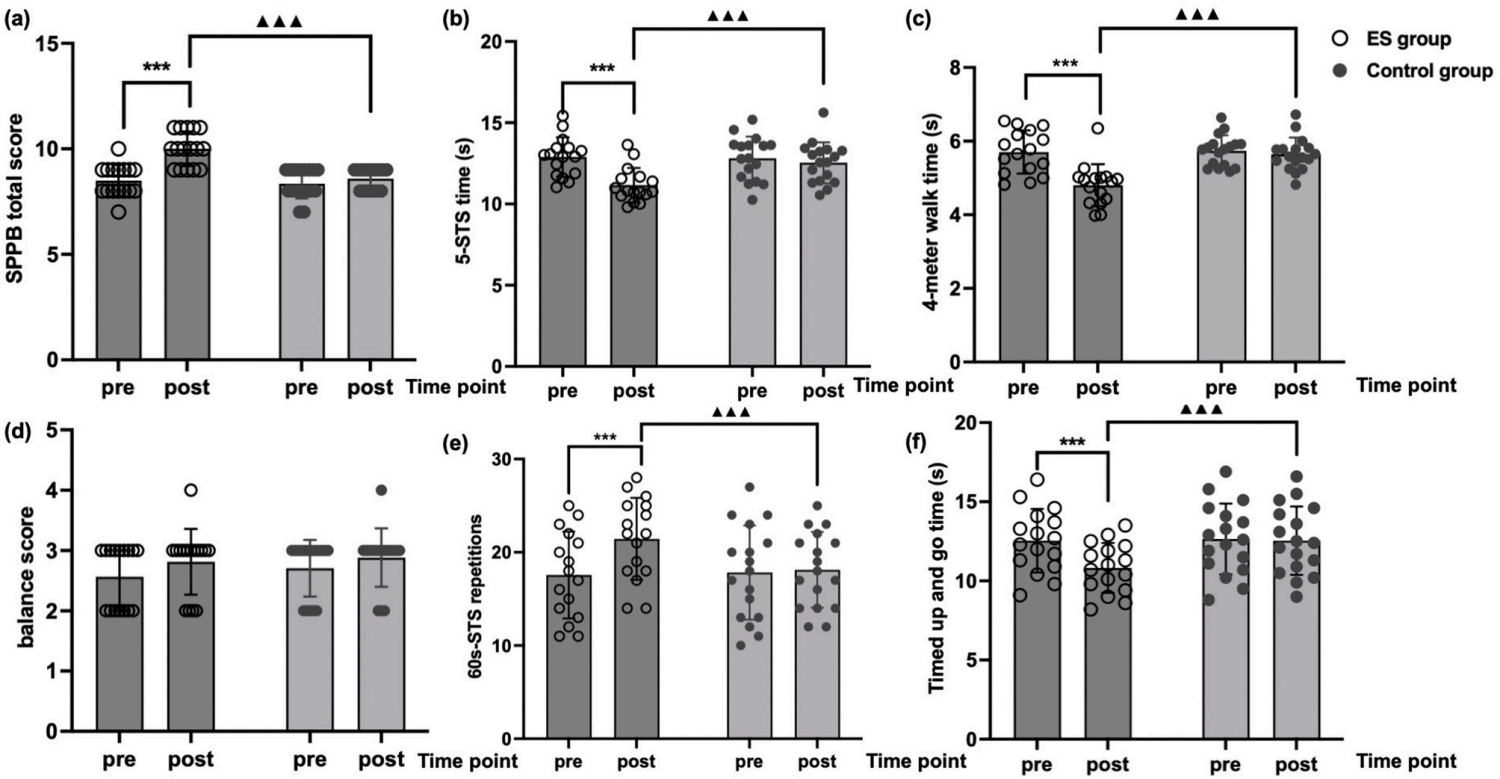
Individual changes in **(a)** total short physical performance battery (SPPB) score, **(b)** five-times sit-to-stand (5-STS) time, **(c)** 4-m walk time, **(d)** balance score, **(e)** 60-s sit-to-stand (60 s-STS) repetitions, and **(f)** timed up and go (TUG) time following the intervention. *** denotes a significant change in the mean value from baseline to follow-up (*p* < 0.001); ▴▴▴ indicates a highly significant time × group interaction (*p* < 0.001).

Figure 2 displays the pre- and post-intervention values for all outcome measures in both groups. The two-way repeated-measures ANOVA revealed a significant interaction between time and group on the total SPPB scores, *F* (1, 31) = 57.90, *p* < 0.0001, partial η^2^ = 0.65, indicating notable differences in the change trends between the experimental and control groups. Simple effects analysis showed that the experimental group exhibited significantly higher post-test scores compared to pre-test scores (*p* < 0.001), confirming a clear intervention effect. In contrast, the control group showed no significant change (*p* = 0.13).

Regarding the SPPB subcomponents, balance scores did not show a significant interaction [*F* (1, 31) = 0.25, *p* = 0.62, partial η^2^ = 0.008], suggesting minimal change across both groups. For the five-STS test, a significant interaction was observed, *F* (1, 31) = 120.8, *p* < 0.0001, partial η^2^ = 0.79. The experimental group showed significant improvement (*p* < 0.001), while the control group showed no significant change (*p* = 0.08), indicating the intervention's effectiveness in improving lower-limb strength. Similarly, for the 4-m walk test, a significant interaction was found, *F* (1, 31) = 83.0, *p* < 0.0001, partial η^2^ = 0.73, with the experimental group demonstrating significant improvement (*p* < 0.001) and the control group showing no significant change (*p* = 0.11), underscoring the intervention's positive effect on walking speed.

In the 60 s-STS test, the interaction between time and group was significant, *F* (1, 31) = 41.33, *p* < 0.0001, partial η^2^ = 0.57. The experimental group showed significant improvement (*p* < 0.001), while the control group showed no significant change (*p* = 0.56), indicating the intervention's effectiveness in improving lower-limb endurance. Similarly, in the TUG time, a significant interaction was found, *F* (1, 31) = 104.6, *p* < 0.0001, partial η^2^ = 0.77. The experimental group showed significant improvement (*p* < 0.001), whereas the control group showed no significant change (*p* = 0.34), highlighting the intervention's positive impact on walking efficiency.

## 4 Discussion

This pilot study investigated the effects of a 4-week home-based “exercise snacking” intervention on muscle function in community-dwelling older adults aged 65–80 years. The main findings indicated that, compared with the control group who maintained their usual daily activities, participants in the ES group demonstrated significant improvements in total scores on the SPPB and in the number of repetitions achieved during the 60 s-STS test, as well as a marked reduction in TUG completion time. These consistent outcomes suggest that brief, accumulated bouts of home-based exercise can produce meaningful improvements in lower-limb strength and endurance, and gait speed in older adults. Collectively, the findings offer preliminary yet compelling evidence supporting “exercise snacking” as a practical and accessible strategy to mitigate age-related declines in physical function among the elderly population.

As a principal assessment tool for evaluating frailty and fall risk in older adults, the SPPB possesses substantial clinical importance (22, 23), therefore, improvements in its score are considered highly meaningful. In this study, the significant improvement in the total SPPB score in the ES group aligns with the findings of Western et al. (24), suggesting that the intervention has produced multiple beneficial effects on lower limb function and walking ability. The underlying physiological mechanisms may involve several interrelated pathways. First, the functional movement of rising from a seated position in the ES protocol heavily depends on both concentric and eccentric contractions of the quadriceps (25, 26). The high-frequency training stimulus, which administered twice daily, 7 days per week, may have preferentially elicited neuromuscular adaptations (27), such as enhanced motor unit recruitment efficiency and improved coordination among synergistic muscles (28). Second, despite the relatively short duration of the intervention, the repeated muscular loading may have activated anabolic signaling pathways (29), including mTOR, thereby stimulating muscle protein synthesis (30). This process likely provided a biological basis for the observed increases in muscle strength and functional capacity. These findings are consistent with the conclusion drawn by Li et al. (31) who reported that high-frequency, low-dose resistance training effectively improves muscle function in older adults.

A detailed analysis of the SPPB sub-tests revealed that the ES intervention significantly improved both the 4-m walk time and the five-STS time, which is consistent with the results of Liang et al. (17) A reduction in 4-m walking time directly reflects increased gait speed, a key indicator of functional capacity and frailty status in older adults (32, 33). The significant decrease in five-STS time more directly indicates gains in lower-limb muscle strength, particularly enhanced quadriceps power and function. Notably, the movement pattern assessed in this test—rapid, repeated transitions from sitting to standing—is a core component of the ES intervention protocol. This high degree of task specificity suggests that the neuromuscular adaptations induced by the intervention were effectively transferred to functionally relevant tasks closely resembling the training stimuli. The study also found no improvement in balance scores, which contrasts with the findings of Liang et al. (17) who observed significant improvements in both balance scores and left leg single-leg stance time. This discrepancy may be attributed to the following factors. First, the ES protocol used in this study primarily emphasized single-joint strength exercises, sit-to-stand from a chair, and marching-in-place practice, which may not have provided sufficient challenge to the static balance system (e.g., single-leg stance). The maintenance and improvement of balance are complex processes that depend on sensory integration (34), vestibular function (35), and coordinated stabilization strategies across multiple joints (36) such as the ankle and hip. These abilities may require more targeted and diverse balance training to be effectively stimulated. Second, given the relatively short intervention period (4 weeks), the neuromuscular system may have preferentially allocated adaptive resources to domains that were directly and strongly stimulated by the intervention—namely, muscle strength and mobility—whereas the more complex balance control system may require a longer training duration to exhibit observable adaptive changes. Finally, the elderly population is highly heterogeneous, with significant variations in the physiological factors contributing to muscle weakness, including neural control capacity, hormone levels, and inflammatory status. Homogeneous, non-personalized exercise programs are insufficient to address the specific needs of individuals (37). Nonetheless, the finding that balance scores in the ES group remained stable from baseline to follow-up and did not differ from those in the control group indicates that the intervention did not adversely affect static balance in older adults, which is an encouraging sign for the safety of home-based exercise programs.

In this study, participants who completed the intervention demonstrated a high overall adherence rate of 82.1%, completing an average of 46 ± 10 exercise sessions, with three participants achieving full compliance. The program's mean acceptability rating was 4.3 out of 5, and qualitative feedback further indicated that participants generally perceived the intervention as feasible, low in burden, and of practical value. Older adults often experience pronounced fatigue, multiple comorbidities, and a fear of engaging in prolonged exercise. The short duration (~9 min per session) and moderate intensity of fragmented exercise substantially reduce both physiological and psychological burdens, thereby enhancing safety during participation (38). By breaking physical activity into manageable “micro-sessions,” this approach encourages the development of regular exercise habits (39) and helps interrupt sedentary behavior patterns driven by fear or low exercise tolerance (40). Safety and adherence appeared to act synergistically during the implementation of the ES program, creating a positive feedback loop. High safety allowed older or frail participants to initiate and sustain engagement without the risk of early withdrawal due to exercise-related discomfort or injury, whereas strong adherence ensured the cumulative training stimulus necessary for functional improvements. In turn, these gains in physical function enhanced participants' confidence and perceived safety during exercise, further encouraging consistent participation and supporting the long-term sustainability of the intervention.

After 4 weeks, participants in the exercise snacking group completed more repetitions, further supporting the conclusions of Perkin (16) and Western (24) that the intervention enhanced fatigue resistance, thereby improving the efficiency of lower limb muscles, particularly the knee and hip extensors (41, 42). This finding holds dual significance. Physiologically, it demonstrates that the cumulative metabolic stress induced by the “exercise snacking” regimen was sufficient to elicit adaptive responses in skeletal muscle (43). Combined with post-intervention interviews with participants, the observed gains in muscular strength and endurance translated into improved performance of everyday activities (44) such as stair climbing and rising from a sofa or toilet—movements that are fundamental for preserving independence and quality of life in older adults (45, 46).

A significant reduction in the TUG completion time is another key finding of this study, consistent with the results of Western et al. (24). In addition to evaluating basic mobility, the TUG test offers a comprehensive assessment of dynamic balance, coordination, and movement confidence in older adults (47, 48). The shortened TUG time observed following the exercise snacking intervention indicates that participants were able to complete the process of standing up and walking more quickly. This improvement may be attributable to marching-in-place exercises and increases in lower-limb strength, as reflected by performance on the five-STS and 60 s-STS tests. Greater muscle strength facilitates faster completion of sit-to-stand transitions (26), while improved dynamic balance helps maintain postural stability during walking and turning (49). From a clinical standpoint, numerous studies have demonstrated that reduced TUG time is strongly associated with a lower risk of falls (50, 51). Therefore, the present findings provide preliminary evidence that even a brief, 4-week ES intervention may represent an effective and low-cost early strategy for fall prevention among older adults.

## 5 Conclusion

This study demonstrates that a 4-week, home-based “exercise snacking” intervention is an innovative, safe, and efficient approach to improve lower-limb strength and endurance, and gait speed in older adults aged 65–80 years. The intervention effectively transforms exercise into an accessible lifestyle practice that older adults can readily adopt and maintain, offering a novel and highly practical strategy to address the challenges of functional decline associated with global population aging.

## 6 Limitations and future directions

Several limitations should be acknowledged and addressed in future research. First, the relatively small sample size (*n* = 33) and short intervention duration (4 weeks) limit the generalizability of the findings and preclude assessment of long-term effects. Second, the mechanisms underlying the observed improvements were not thoroughly investigated. The absence of objective measures, such as isokinetic muscle strength testing, dual-energy X-ray absorptiometry (DEXA), or surface electromyography, it restricted the ability to quantify deeper changes in muscle strength, body composition, and neuromuscular activation patterns, rendering mechanistic interpretations largely speculative.

Future studies should integrate multi-omics approaches could help elucidate the molecular mechanisms by which exercise snacking influences neuromuscular function, muscle metabolism, and systemic inflammation. Furthermore, research should explore individualized dose-response relationships to determine optimal session intensity, daily frequency, and tailored exercise prescriptions suited to varying functional capacities among older adults.

## Data Availability

The original contributions presented in the study are included in the article/Supplementary material, further inquiries can be directed to the corresponding author.

## References

[1] Bloom DE Canning D Lubet A. Global population aging: facts, challenges, solutions & perspectives. Daedalus. (2015) 144:80–92. doi: 10.1162/DAED_a_00332

[2] Navaneetham K Arunachalam D. Global population aging, 1950–2050. Handbook of Aging, Health and Public Policy: Perspectives from Asia. Singapore: Springer (2025). p. 99–116. doi: 10.1007/978-981-99-7842-7_154

[3] Walston JD. Sarcopenia in older adults. Curr Opin Rheumatol. (2012) 24:623–7. doi: 10.1097/BOR.0b013e328358d59b 22955023 PMC4066461

[4] Bachettini NP Bielemann RM Barbosa-Silva TG Menezes AMB Tomasi E Gonzalez MC. Sarcopenia as a mortality predictor in community-dwelling older adults: a comparison of the diagnostic criteria of the European working group on sarcopenia in older people. Eur J Clin Nutr. (2020) 74:573–80. doi: 10.1038/s41430-019-0508-8 31586126

[5] Granic A Sayer AA Robinson SM. Dietary patterns, skeletal muscle health, and sarcopenia in older adults. Nutrients. (2019) 11:745. doi: 10.3390/nu11040745 30935012 PMC6521630

[6] Meier NF Lee D-c. Physical activity and sarcopenia in older adults. Aging Clin Exp Res. (2020) 32:1675–87. doi: 10.1007/s40520-019-01371-8 31625078

[7] de Labra C Guimaraes-Pinheiro C Maseda A Lorenzo T Millán-Calenti JC. Effects of physical exercise interventions in frail older adults: a systematic review of randomized controlled trials. BMC Geriatr. (2015) 15:154. doi: 10.1186/s12877-015-0155-4 26626157 PMC4667405

[8] Liu C-j Chang W-P de Carvalho IA Savage KE Radford LW Thiyagarajan JA. Effects of physical exercise in older adults with reduced physical capacity: meta-analysis of resistance exercise and multimodal exercise. Int J Rehabil Res. (2017) 40:303–14. doi: 10.1097/MRR.0000000000000249 29023317

[9] Burton E Lewin G Pettigrew S Hill A-M Bainbridge L Farrier K . Identifying motivators and barriers to older community-dwelling people participating in resistance training: a cross-sectional study. J Sports Sci. (2017) 35:1523–32. doi: 10.1080/02640414.2016.1223334 27559917

[10] Burton E Farrier K Lewin G Pettigrew S Hill A-M Airey P . Motivators and barriers for older people participating in resistance training: a systematic review. J Aging Phys Act. (2017) 25:311–24. doi: 10.1123/japa.2015-0289 27620535

[11] Jones MD Clifford BK Stamatakis E Gibbs MT. Exercise snacks and other forms of intermittent physical activity for improving health in adults and older adults: a scoping review of epidemiological, experimental and qualitative studies. Sports Med. (2024) 54:813–35. doi: 10.1007/s40279-023-01983-1 38190022

[12] Gokal K Amos-Hirst R Moakes CA Sanders JP Esliger DW Sherar LB . Views of the public about snacktivity: a small changes approach to promoting physical activity and reducing sedentary behaviour. BMC Public Health. (2022) 22:618. doi: 10.1186/s12889-022-13050-x 35351075 PMC8964250

[13] Moore DR Williamson EP Hodson N Estafanos S Mazzulla M Kumbhare D . Walking or body weight squat “activity snacks” increase dietary amino acid utilization for myofibrillar protein synthesis during prolonged sitting. J Appl Physiol (1985). (2022) 133:777–85. doi: 10.1152/japplphysiol.00106.2022 35952344

[14] Francois ME Baldi JC Manning PJ Lucas SJ Hawley JA Williams MJ . ‘Exercise snacks' before meals: a novel strategy to improve glycaemic control in individuals with insulin resistance. Diabetologia. (2014) 57:1437–45. doi: 10.1007/s00125-014-3244-6 24817675

[15] Sullivan AB Covington E Scheman J. Immediate benefits of a brief 10-minute exercise protocol in a chronic pain population: a pilot study. Pain Medicine. (2010) 11:524–9. doi: 10.1111/j.1526-4637.2009.00789.x 20113415

[16] Perkin OJ McGuigan PM Stokes KA. Exercise snacking to improve muscle function in healthy older adults: a pilot study. J Aging Res. (2019) 2019:7516939. doi: 10.1155/2019/7516939 31687210 PMC6794984

[17] Liang IJ Perkin OJ Williams S McGuigan PM Thompson D Western MJ. The efficacy of 12-week progressive home-based strength and Tai-Chi exercise snacking in older adults: a mixed-method exploratory randomised control trial. J Frailty Aging. (2024) 13:572–81. doi: 10.14283/jfa.2024.32 39574284

[18] Liang IJ Perkin OJ McGuigan PM Thompson D Western MJ. Feasibility and acceptability of home-based exercise snacking and Tai Chi snacking delivered remotely to self-isolating older adults during COVID-19. J Aging Phys Act. (2022) 30:33–43. doi: 10.1123/japa.2020-0391 34157675

[19] Fyfe JJ Dalla Via J Jansons P Scott D Daly RM. Feasibility and acceptability of a remotely delivered, home-based, pragmatic resistance ‘exercise snacking' intervention in community-dwelling older adults: a pilot randomised controlled trial. BMC Geriatr. (2022) 22:521. doi: 10.1186/s12877-022-03207-z 35751032 PMC9233333

[20] Jansons P Dalla Via J Daly RM Fyfe JJ Gvozdenko E Scott D. Delivery of home-based exercise interventions in older adults facilitated by amazon alexa: a 12-week feasibility trial. J Nutr Health Aging. (2022) 26:96–102. doi: 10.1007/s12603-021-1717-0 35067710 PMC12275629

[21] Guralnik JM Simonsick EM Ferrucci L Glynn RJ Berkman LF Blazer DG . A short physical performance battery assessing lower extremity function: association with self-reported disability and prediction of mortality and nursing home admission. J Gerontol. (1994) 49:M85–94. doi: 10.1093/geronj/49.2.M85 8126356

[22] de Fátima Ribeiro Silva C Ohara DG Matos AP Pinto ACPN Pegorari MS. Short physical performance battery as a measure of physical performance and mortality predictor in older adults: a comprehensive literature review. Int J Environ Res Public Health. (2021) 18:10612. doi: 10.3390/ijerph182010612 34682359 PMC8535355

[23] Treacy D Hassett L. The short physical performance battery. J Physiother. (2018) 64:61. doi: 10.1016/j.jphys.2017.04.002 28645532

[24] Western MJ Welsh T Keen K Bishop V Perkin OJ. Exercise snacking to improve physical function in pre-frail older adult memory clinic patients: a 28-day pilot study. BMC Geriatrics. (2023) 23:471. doi: 10.1186/s12877-023-04169-6 37542234 PMC10403822

[25] Ganea R Paraschiv-Ionescu A Büla C Rochat S Aminian K. Multi-parametric evaluation of sit-to-stand and stand-to-sit transitions in elderly people. Med Eng Phys. (2011) 33:1086–93. doi: 10.1016/j.medengphy.2011.04.015 21601505

[26] Fujita E Taaffe DR Yoshitake Y Kanehisa H. Repeated sit-to-stand exercise enhances muscle strength and reduces lower body muscular demands in physically frail elders. Exp Gerontol. (2019) 116:86–92. doi: 10.1016/j.exger.2018.12.016 30593854

[27] Conlon JA Newton RU Tufano JJ Peñailillo LE Banyard HG Hopper AJ . The efficacy of periodised resistance training on neuromuscular adaptation in older adults. Eur J Appl Physiol. (2017) 117:1181–94. doi: 10.1007/s00421-017-3605-1 28401310

[28] Granacher U Gruber M Gollhofer A. Resistance training and neuromuscular performance in seniors. Int J Sports Med. (2009) 30:652–7. doi: 10.1055/s-0029-1224178 19569007

[29] Lamas L Aoki MS Ugrinowitsch C Campos G Regazzini M Moriscot AS . Expression of genes related to muscle plasticity after strength and power training regimens. Scand J Med Sci Sports. (2010) 20:216–25. doi: 10.1111/j.1600-0838.2009.00905.x 19422645

[30] Coffey VG Reeder DW Lancaster GI Yeo WK Febbraio MA Yaspelkis III BB . Effect of high-frequency resistance exercise on adaptive responses in skeletal muscle. Med Sci Sports Exerc. (2007) 39:2135–44. doi: 10.1249/mss.0b013e31815729b6 18046184

[31] Li Q Huang F Cheng Y Dai Y Lin Z Lin Z . Does high-frequency resistance exercise offer additional benefits to older adults? Learnings from a randomized controlled trial. BMC Sports Sci Med Rehabil. (2024) 16:186. doi: 10.1186/s13102-024-00975-6 39243106 PMC11378542

[32] Nguyen AT Nguyen HTT Nguyen HTT Nguyen TX Nguyen TN Nguyen TTH . Walking speed assessed by 4-meter walk test in the community-dwelling oldest old population in Vietnam. Int J Environ Res Public Health. (2022) 19:9788. doi: 10.3390/ijerph19169788 36011423 PMC9407834

[33] Peters DM Fritz SL Krotish DE. Assessing the reliability and validity of a shorter walk test compared with the 10-meter walk test for measurements of gait speed in healthy, older adults. J Geriatr Phys Ther. (2013) 36:24–30. doi: 10.1519/JPT.0b013e318248e20d 22415358

[34] Pu F Sun S Wang L Li Y Yu H Yang Y . Investigation of key factors affecting the balance function of older adults. Aging Clin Exp Res. (2015) 27:139–47. doi: 10.1007/s40520-014-0253-8 25182452

[35] Marchetti GF Whitney SL Redfern MS Furman JM. Factors associated with balance confidence in older adults with health conditions affecting the balance and vestibular system. Arch Phys Med Rehabil. (2011) 92:1884–91. doi: 10.1016/j.apmr.2011.06.015 22032223 PMC4886544

[36] Menz HB Morris ME Lord SR. Foot and ankle characteristics associated with impaired balance and functional ability in older people. J Gerontol A Biol Sci Med Sci. (2005) 60:1546–52. doi: 10.1093/gerona/60.12.1546 16424286

[37] Alexe D Saha S Choudhary P Alexe C Choudhary S Tohănean D. Exercise snacks as a strategy to interrupt sedentary behavior: a systematic review of health outcomes and feasibility. Healthcare. (2025) 13:3216. doi: 10.3390/healthcare13243216 41464286 PMC12732512

[38] Weston KL Little JP Weston M McCreary S Kitchin V Gill A . Application of exercise snacks across youth, adult and clinical populations: a scoping review. Sports Med Open. (2025) 11:27. doi: 10.1186/s40798-025-00829-6 40102333 PMC11920532

[39] Rodríguez MÁ Quintana-Cepedal M Cheval B Thøgersen-Ntoumani C Crespo I Olmedillas H. Effect of exercise snacks on fitness and cardiometabolic health in physically inactive individuals: systematic review and meta-analysis. Br J Sports Med. (2025). doi: 10.1136/bjsports-2025-110027. [Epub ahead of print]. 41057224

[40] Wang T Laher I Li S. Exercise snacks and physical fitness in sedentary populations. Sports Med Health Science. (2025) 7:1–7. doi: 10.1016/j.smhs.2024.02.006 39649791 PMC11624330

[41] Glenn JM Gray M Binns A. Relationship of sit-to-stand lower-body power with functional fitness measures among older adults with and without sarcopenia. J Geriatr Phys Ther. (2017) 40:42–50. doi: 10.1519/JPT.0000000000000072 26428899

[42] Park TS Shin M-J. Comprehensive assessment of lower limb function and muscle strength in sarcopenia: insights from the sit-to-stand test. Ann Geriatr Med Res. (2024) 28:1. doi: 10.4235/agmr.23.0205 38325818 PMC10982452

[43] Nuzzo JL Pinto MD Kirk BJC Nosaka K. Resistance exercise minimal dose strategies for increasing muscle strength in the general population: an overview. Sports Med. (2024) 54:1139–62. doi: 10.1007/s40279-024-02009-0 38509414 PMC11127831

[44] Tyldesley-Marshall N Greenfield SM Parretti HM Gokal K Greaves C Jolly K . Snacktivity to promote physical activity: a qualitative study. Int J Behav Med. (2022) 29:553–64. doi: 10.1007/s12529-021-10040-y 34782996 PMC8592280

[45] Holviala J Kraemer W Sillanpää E Karppinen H Avela J Kauhanen A . Effects of strength, endurance and combined training on muscle strength, walking speed and dynamic balance in aging men. Eur J Appl Physiol. (2012) 112:1335–47. doi: 10.1007/s00421-011-2089-7 21796409

[46] Kim HJ Hong YS. An effect of muscle strength training program on muscle strength, muscle endurance, instrumental activities of daily living and quality of life in the institutionalized elderly. J Korean Community Nurs. (1995) 6:55–73.

[47] BeauchetO FantinoB AllaliG MuirS Montero-OdassoM AnnweilerC. Timed up and go test and risk of falls in older adults: a systematic review. J Nutr Health Aging. (2011) 15:933–8. doi: 10.1007/s12603-011-0062-022159785

[48] Barry E Galvin R Keogh C Horgan F Fahey T. Is the timed up and go test a useful predictor of risk of falls in community dwelling older adults: a systematic review and meta-analysis. BMC Geriatr. (2014) 14:14. doi: 10.1186/1471-2318-14-14 24484314 PMC3924230

[49] Ng S. Balance ability, not muscle strength and exercise endurance, determines the performance of hemiparetic subjects on the timed-sit-to-stand test. Am J Phys Med Rehabil. (2010) 89:497–504. doi: 10.1097/PHM.0b013e3181d3e90a 20216059

[50] Schoene D Wu SMS Mikolaizak AS Menant JC Smith ST Delbaere K . Discriminative ability and predictive validity of the timed up and go test in identifying older people who fall: systematic review and meta-analysis. J Am Geriatr Soc. (2013) 61:202–8. doi: 10.1111/jgs.12106 23350947

[51] Shumway-Cook A Brauer S Woollacott M. Predicting the probability for falls in community-dwelling older adults using the timed up & go test. Phys Ther. (2000) 80:896–903. doi: 10.1093/ptj/80.9.896 10960937

