# Supplementary material for: Feasibility and acceptability of a remotely delivered, home-based “exercise snacking” to improve physical function in community-dwelling older adults: a 28-day pilot study
Source: Front Med (Lausanne). 2026 Jan 23;13:1755508. doi: 10.3389/fmed.2026.1755508 (PMC12879047; doi:10.3389/fmed.2026.1755508)
Supplement: Supplementary file 1 [file Table_1.DOCX]

**Figure 1-5 exercise snacking movements**

Before you begin

Please use a sturdy chair with armrests, that does not wobble, has no wheels, and is approximately 40 cm high.

1. **Sit-to-stand (STS) from a chair**
2. Sit in the middle of the chair. Place both feet flat on the floor, hip-width apart, with your lower legs vertical. Cross your arms over your chest or hold your arms straight out in front of you.
3. Use the strength of your thighs to stand up from the chair. If this is too difficult, you may push on the armrests or the seat with your hands to help you stand.
4. Slowly sit back down until the back of your hamstrings are fully in contact with the seat of the chair.
5. **
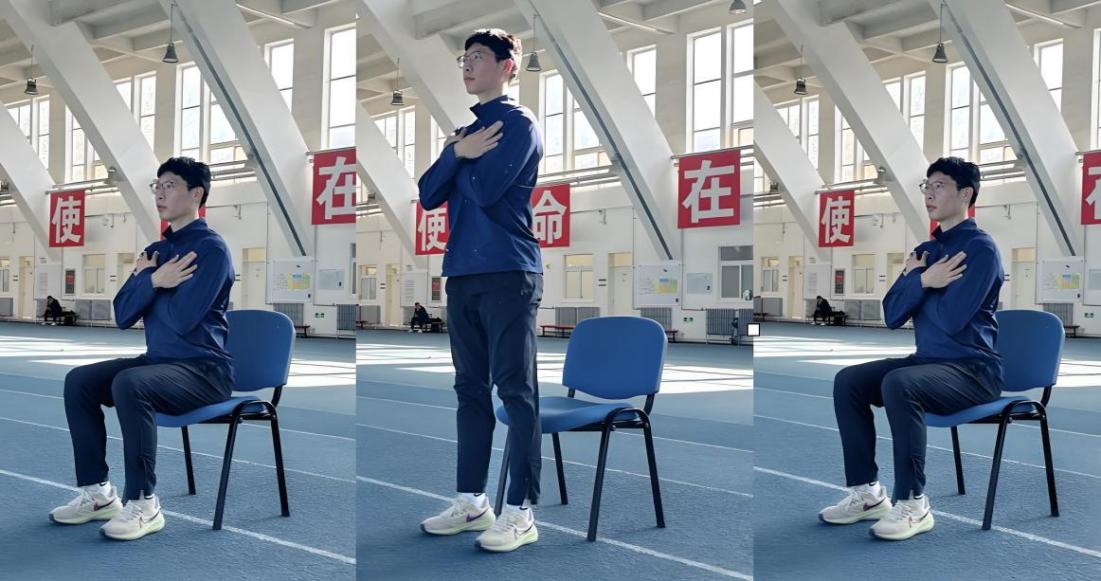
Seated knee extensions with alternating legs**
6. Sit in the middle of the chair. Place both feet flat on the floor, hip-width apart, with your lower legs vertical. Rest your hands naturally on your thighs.
7. Keep one foot firmly on the floor. With the other leg, tighten your thigh muscles (quadriceps) and straighten your knee until your thigh and lower leg are roughly parallel to the floor. If this is too difficult, you may straighten only at the knee as far as you comfortably can.
8. **
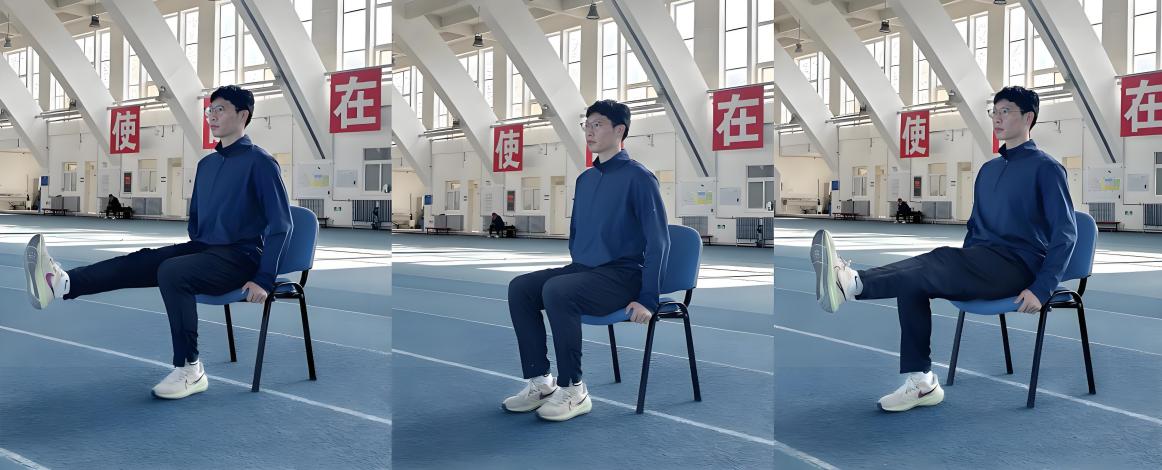
**Return that leg to the starting position with both feet flat on the floor, then repeat the movement with the other leg.
9. **Standing knee bends with alternating legs**
10. Stand behind the chair with both feet flat on the floor. Hold the back of the chair with one hand for support.
11. Keep one foot firmly on the floor. With the other leg, bend your knee so that your heel moves toward your buttock, keeping the knee pointing straight down. If the movement is too challenging, you may flex your knees within a comfortable range according to your individual capacity.
12. Return that leg to the starting position with both feet flat on the floor, then repeat the movement with the other leg.
13. **
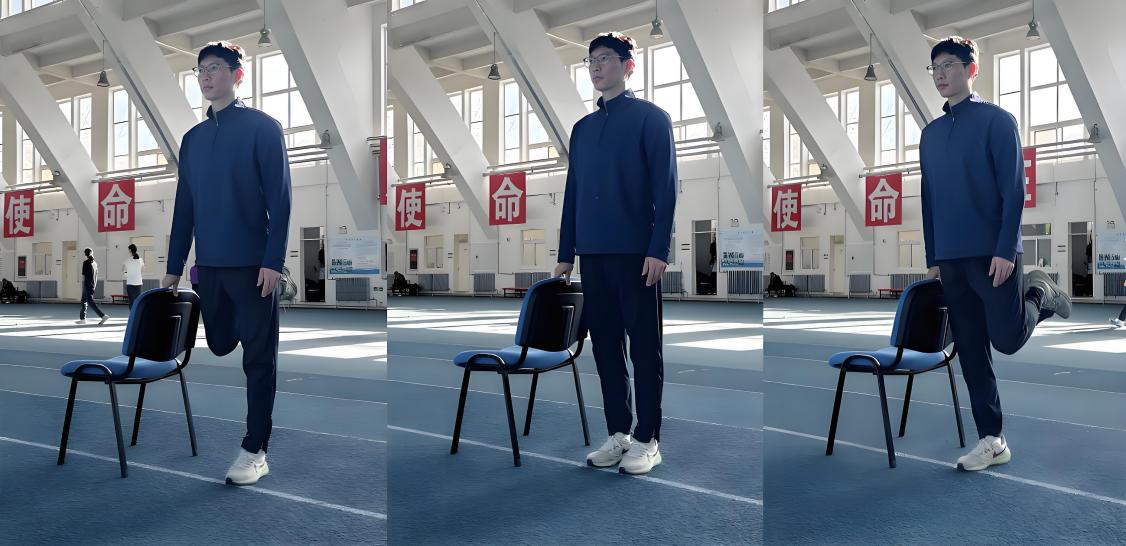
Marching in place**
14. Stand behind the chair with both feet flat on the floor. Hold the back of the chair with one hand for support.
15. Keep one foot firmly on the floor. Lift the other leg so that your thigh is roughly parallel to the floor.
16. Lower the leg and repeat with the opposite leg, alternating legs in a marching motion.

**
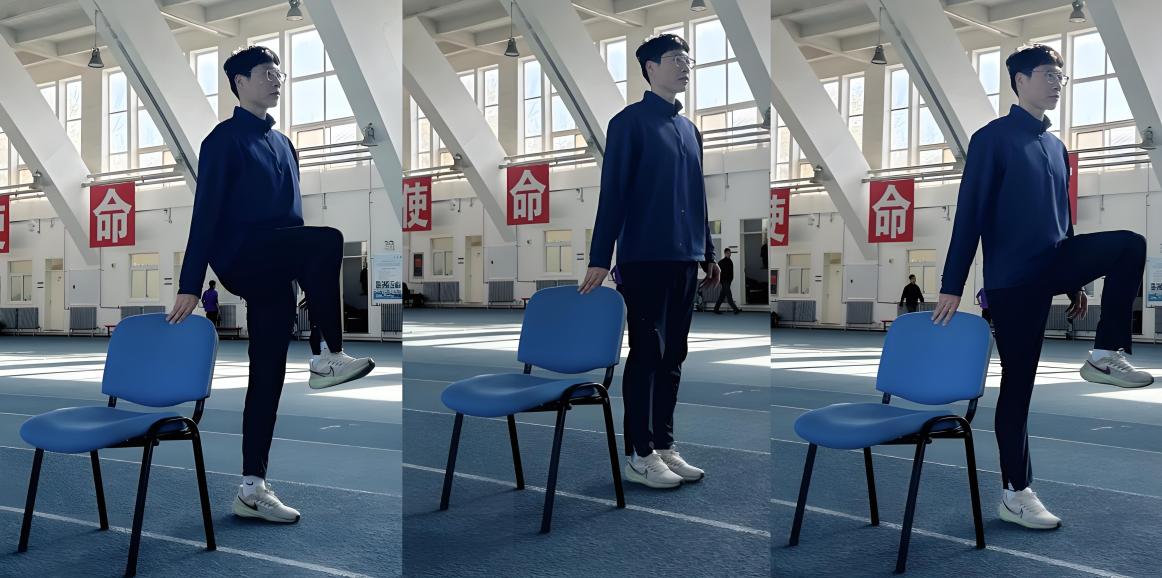
**

1. **Standing calf raises**
2. Stand behind the chair with both feet flat on the floor. Hold the back of the chair with one hand for support.
3. Raise your heels as high as you comfortably can so that you are standing on the balls of your feet.
4. Slowly lower your heels back down until both feet are flat on the floor again.

**
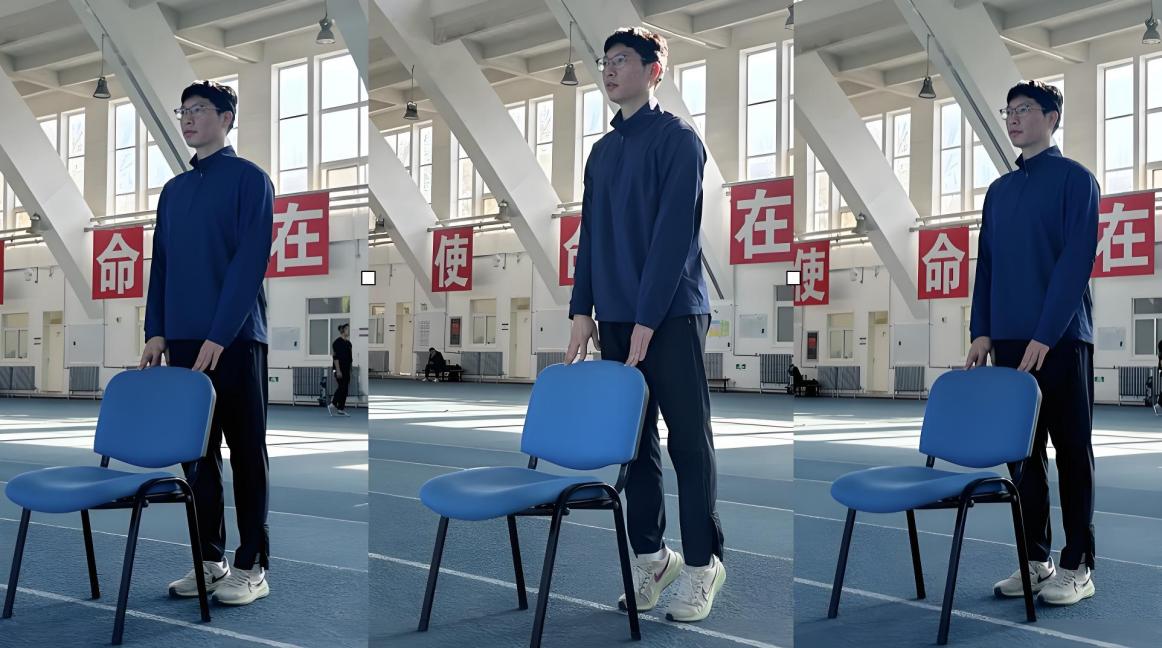
**

**Table 1 Participant quotes based on the semi-structured interviews are presented along with individual characteristics [sex, age]**

| **Response theme** | **Example participant responses** |
| --- | --- |
| Integration Into Daily Life | "(The exercise program) didn't get in the way of my daily routine, so sticking to it was pretty easy." **[Male, 69]**  "What I liked was that I could exercise even while watching TV.” **[Female, 72]** |
| Feasibility and Ease of Implementation | "I liked that the exercises were simple, quick to finish, and spread throughout the day. They were easy to do and didn’t need any equipment or setup.” **[Female, 67]**  "The exercise program basically made me set aside a bit of time each day to exercise. Otherwise, I usually just keep pushing it off and telling myself I’ll get to it later.” **[Male, 77]** |
| Relevance to Health Needs | “The program also reminded me that I really need to do more of these kinds of exercises if I want to improve my balance and muscle tone.” **[Female, 75]**  “I liked that the program was short but still focused on the important stuff—like balance, strength, and agility.” **[Male, 79]**  “Doing the exercises regularly has helped improve my strength and flexibility a bit.” **[Female, 66]** |
| Increased Motivation and Exercise Engagement | “I know I should be more active, so having to do the exercises twice a day was actually good for me. My family was happy about it too!” **[Male, 69]**  “I liked discovering how much exercise I was actually capable of, since I usually just take a walk every day.” **[Female, 73]**  “This program pushed me to put in more effort. I feel fitter now—thanks for the experience.” **[Female, 74]** |
| Need for Individualized Adjustments | “This series is really good. The exercises and the pacing are perfect for helping seniors like me get back into a workout routine.” **[Male, 72]**  “The ‘standing knee bends’ exercise makes my hamstrings cramp—probably because that area’s a bit weak for me. I’d love to see some easier, more basic alternatives.” **[Female, 79]**  “The exercises are very simple and easy to follow, but I hope there will be some more advanced options too so I can challenge myself and get better muscle stimulation.” **[Male, 65]** |
| Suggestions for Program Enhancement | “I’d like to add more variety to my workouts. I have a bit of a hunchback, so I especially want to strengthen my back muscles.” **[Male, 76]**  “There are five exercises in this routine, but I’d love to mix it up a bit more—maybe add some arm exercises so I can even work out while watching TV.” **[Female, 69]**  “I think the program is great. It’d be even better with some cardio, though. Whenever my partner and I go for a brisk walk, I’m always out of breath while they’re totally fine!” **[Female, 71]** |
